# Enhanced Adsorption of Bisphenol a on Lignin-Derived Biochars: Role of Thermal and Phosphoric Acid Activation in Surface Functionalization and Mechanism

**DOI:** 10.3390/polym17233159

**Published:** 2025-11-27

**Authors:** Francisco Flores-Céspedes, Iván González-Fernández, Manuel Fernández-Pérez, Luis García-Fuentes

**Affiliations:** Department of Chemistry and Physics, University of Almería, Crta. Sacramento s/n, 04120 Almería, Spain; ivan.gonzalez@ciqso.uhu.es (I.G.-F.); mfernand@ual.es (M.F.-P.); lgarcia@ual.es (L.G.-F.)

**Keywords:** bisphenol A, lignin-derived biochar, phosphoric acid activation, adsorption

## Abstract

This study investigated the adsorption behavior of bisphenol A (BPA) onto a series of thermally and acid-activated biochars to elucidate the relationship between the surface properties and adsorption performance. Characterization analyses (FTIR, SEM, BET, elemental composition, and PZC) revealed that phosphoric acid activation significantly increased the surface area, pore development, and oxygen/phosphate functionalization, lowering the point of zero charge (PZC = 1.3) and enhancing the surface acidity. The kinetic data fitted well to the pseudo-second-order model, indicating a chemisorption-controlled process, while the equilibrium data were best described by the Langmuir model, with a maximum adsorption capacity (*q_m_* = 262.28 ± 14.3 mg·g^−1^) for the acid-activated biochar (LB_450_-H_3_PO_4_). Thermodynamic analysis confirmed that the adsorption process is spontaneous and endothermic (ΔH° > 0), with a highly favorable entropy contribution. The effects of solution pH, adsorbent dosage, initial BPA concentration, and temperature demonstrated optimal removal under acidic to neutral conditions and moderate dosage (0.2 g·L^−1^). Overall, the findings highlight that phosphoric acid activation effectively enhances surface functionality and charge properties, transforming biochar into a highly efficient and sustainable adsorbent for the removal of phenolic contaminants from aqueous solutions.

## 1. Introduction

The rapid expansion of industrial manufacturing and the increasing global demand for plastic-based products have contributed to the proliferation of synthetic organic chemicals in the environment. Among these, emerging contaminants (ECs) have become a category of growing concern. ECs comprise a diverse group of synthetic or naturally occurring compounds that are not routinely monitored but may pose significant ecological and human health risks due to their persistence and biological activity [1]. These include pharmaceuticals and personal care products (PPCPs), endocrine-disrupting compounds (EDCs), per- and polyfluoroalkyl substances (PFAs), micro- and nanoplastics, and various industrial additives such as bisphenol A (BPA) [2]. Governments and researchers have increasingly emphasized the removal and recovery of these pollutants to restore the environmental quality and protect public health [3].

BPA is one of the most representative and problematic ECs due to its extensive use as a monomer in the production of polycarbonate plastics and epoxy resins, which are widely utilized in food and beverage containers, thermal paper, medical devices, and metal can linings [1,4]. Under conditions of heat or mechanical stress, BPA can leach from polymeric materials into the surrounding environment and subsequently enter aquatic systems or food chains [3]. Once released, BPA acts as an endocrine-disrupting chemical, capable of mimicking natural hormones such as estrogen, disrupting endocrine signaling pathways and causing physiological dysfunctions [2]. Chronic exposure has been linked to reproductive toxicity, metabolic and developmental disorders, and an increased risk of hormone-related cancers [5,6].

Due to its widespread distribution, chemical stability, and biological activity at trace levels, BPA exemplifies the challenges associated with plastic-associated emerging pollutants. Despite partial regulatory restrictions on BPA usage in certain consumer goods, environmental contamination remains significant, primarily due to legacy products and the limited efficiency of conventional wastewater treatment systems [1]. Traditional treatment methods, such as advanced oxidation processes, photodegradation, and membrane filtration, often suffer from drawbacks, including incomplete mineralization, high operational costs, and the formation of toxic by-products. In contrast, adsorption has emerged as a promising approach for BPA removal, offering advantages such as operational simplicity, low cost, and minimal secondary pollution.

Among the range of available adsorbents, biochar, a carbon-rich, porous material derived from the pyrolysis of biomass under oxygen-limited conditions, has attracted substantial attention for its application in environmental remediation [4,7]. Its physicochemical characteristics, such as surface area, pore distribution, functional groups, and hydrophobicity, can be tuned by adjusting the feedstock type and pyrolysis temperature, allowing targeted optimization for pollutant adsorption [1]. Furthermore, biochar production contributes to circular economic strategies by transforming agricultural and industrial waste into high-value sorbents [4].

Lignin, the second most abundant biopolymer on Earth after cellulose, is an underutilized by-product of the pulp, paper, and biorefinery industries. Owing to its aromatic structure, high carbon content, and functional group diversity, lignin represents a promising precursor for engineered biochar production [8,9,10]. Converting lignin into biochar not only valorizes industrial waste streams but also yields materials with surface characteristics favorable for the adsorption of aromatic organic pollutants such as BPA [11]. Several studies have demonstrated that lignin-derived biochar exhibits a strong affinity for aromatic contaminants through π–π electron donor–acceptor interactions, hydrogen bonding, and electrostatic attraction [5,12]. Moreover, the sorption performance of lignin biochar can be further enhanced via chemical activation or heteroatom doping, improving the surface area and binding site availability [1,13]. Phosphoric acid (H_3_PO_4_) was selected as the activating agent due to its dual role as both a dehydrating and crosslinking catalyst. Unlike alkaline (KOH, NaOH) or metallic (ZnCl_2_) activators, H_3_PO_4_ promotes the formation of stable phosphate and polyphosphate linkages during pyrolysis, which prevents excessive shrinkage of the carbon framework and facilitates the development of a porous structure. Moreover, phosphoric acid introduces oxygenated and phosphorus-containing surface groups that enhance the acidity and polarity of the biochar, improving its affinity for aromatic and polar organic pollutants such as BPA. In addition, H_3_PO_4_ is less corrosive, easier to handle, and more environmentally benign than other commonly used chemical activators [13].

In this context, the present study aims to investigate a direct comparison between thermally and phosphoric-acid-activated lignin-derived biochars for the adsorption of BPA from aqueous media. Unlike most previous studies, our systematically analyzes how the activation route affects surface chemistry, charge properties, and adsorption mechanisms. Specifically, the objectives are to: (i) synthesize and characterize biochar prepared from lignin under controlled pyrolysis conditions; (ii) evaluate the influence of operational parameters such as pH, contact time, and initial BPA concentration on adsorption performance; and (iii) elucidate the adsorption mechanisms through kinetic, equilibrium, and thermodynamic modeling. This comparative approach provides new insights into how chemical activation controls the interplay between surface functionalization and adsorption efficiency, advancing the design of sustainable, high-performance lignin-based adsorbents for endocrine-disrupting pollutants.

## 2. Materials and Methods

### 2.1. Chemicals and Reagents

Bisphenol A (BPA, C_15_H_16_O_2_; ≥99% purity) was purchased from Sigma–Aldrich (St. Louis, MO, USA). Kraft lignin (Indulin AT, L) was supplied by Westvaco Corporation (Charleston, SC, USA) and was used as the biochar precursor. Phosphoric acid (H_3_PO_4_, ≥85%, Merck KGaA, Darmstadt, Germany), sodium hydroxide (NaOH, ≥99%, Riedel-de Haën, Seelze, Germany), and hydrochloric acid (HCl, ≥37%, Honeywell, Seelze, Germany) were used as activating or conditioning agents. Acetonitrile (CH_3_CN, ≥99.9%, Honeywell, Chromasolv for HPLC) was used as the organic component of the mobile phase. All solutions were prepared with deionized water (resistivity ≥ 18.2 MΩ cm).

### 2.2. Preparation of the Biochar Adsorbents

#### 2.2.1. Thermal Activation

Thermally activated lignin-based biochars were prepared using a Heraeus M-110 muffle furnace (Heraeus Holding GmbH, Hanau, Germany). Raw lignin (<0.5 mm particle size) was dried and then subjected to heat treatment under limited oxygen conditions. Approximately 100 g of lignin was placed in porcelain crucibles and heated at different temperatures (110, 250, 350, and 450 °C) for 1 h under limited oxygen conditions. The heat treatments were carried out in a muffle furnace under static air without forced gas flow. The lignin was placed in a porcelain crucible, and the oxygen available inside the furnace was progressively consumed during heating, so that the samples were effectively treated under oxygen-limited conditions. The partial pressure of O_2_ was not monitored directly, and therefore no quantitative value can be reported. After heating, the samples were transferred to a desiccator to cool to room temperature, preventing uncontrolled oxidation.

The cooled samples were ground using an agate mortar and a Retsch S100 ball mill (Retsch GmbH, Hann, Germany), then sieved to a particle size ≤ 0.2 mm using a Retsch AS 200 analytical sieve shaker (Retsch GmbH). The resulting biochars were designated LB_110_, LB_250_, LB_350_, and LB_450_, corresponding to their preparation temperatures. All samples were stored in polypropylene containers within a desiccator until further use.

#### 2.2.2. Chemical Activation

Chemical activation was performed using phosphoric acid (H_3_PO_4_). One hundred grams of crude lignin was mixed with concentrated H_3_PO_4_ at a 1:1.4 (*w*/*w*) ratio, corresponding to 96.3 mL of acid. The mixture was homogenized into a paste and left to stand for 1 h at room temperature to ensure complete impregnation.

Subsequently, pyrolysis was conducted at 450 °C for 1 h in a limited-oxygen atmosphere. The resulting solid was washed repeatedly with distilled water until the pH of the filtrate stabilized (pH ≈ 1.9; ~20 L of wash water used). The washed biochar was dried at 110 °C for 1 h, ground, sieved to ≤0.2 mm, and stored as described above. The chemically activated sample was denoted LB_450_–H_3_PO_4_.

The activation temperature of 450 °C was selected as a compromise between achieving effective surface activation and minimizing thermal degradation of lignin, providing an optimal balance for stable biochar structure and adsorption performance.

### 2.3. Characterization of the Biochars

#### 2.3.1. Elemental Analysis

The elemental composition (C, H, N, S) was determined using an Elementar Vario Micro CHNS analyzer (Elementar Analysensysteme GmbH, Langenselbold, Germany). The samples (2–6 mg) were combusted at 1200 °C, and the evolved gases were separated and quantified using a thermal conductivity detector (TCD).

#### 2.3.2. Fourier Transform Infrared Spectroscopy (FTIR)

The surface functional groups were identified using a Bruker ALPHA II FTIR spectrometer equipped with an ATR accessory. Spectra were recorded in the 4000–600 cm^−1^ range with a spectral resolution of 4 cm^−1^. The number of scans used was 64 and the arithmetic average was calculated to generate the final spectra.

#### 2.3.3. Scanning Electron Microscopy

Surface morphology was examined by field emission scanning electron microscopy (FESEM) using a Zeiss Sigma 300 VP microscope (Carl Zeiss Microscopy GmbH, Oberkochen, Alemania). Samples were coated with a 5 nm gold layer under high vacuum. Images were obtained at 5 kV accelerating voltage, 9 mm working distance, and magnifications of 200×, and 1000×.

#### 2.3.4. Specific Surface Area

The nitrogen adsorption–desorption isotherms were measured at 77 K using a Micromeritics Gemini VII 2390 surface analyzer (Micromeritics Instrument Corporation, Norcross, GA, US) after degassing the samples in a Micromeritics FlowPrep 060 unit (Micromeritics Instrument Corporation). The specific surface areas were calculated according to the Brunauer–Emmett–Teller (BET) model.

#### 2.3.5. Point of Zero Charge (PZC)

The point of zero charge was determined by the pH-drift method. A 0.01 g sample of LB_450_–H_3_PO_4_ biochar was added to 50 mL of 0.1 M NaCl solution, and the initial pH was adjusted from 1 to 10 using HCl or NaOH. After shaking for 24 h at 25 °C (200 rpm), the final pH was measured using a Crison GLP 21 pH meter (Crison Instruments, S.A., Barcelona, Spain). The PZC was taken as the pH where ΔpH = 0.

### 2.4. Analytical Determination of the BPA

BPA quantification was performed by high-performance liquid chromatography (HPLC) using a Beckman Coulter system (Beckman Coulter, Inc., Brea, CA, USA) comprising a System Gold 126 gradient pump, a System Gold 508 autosampler, and a System Gold 168 diode array detector (λ = 228 nm). Separation was achieved on a C18 column (150 × 3.9 mm) using a 50:50 (*v*/*v*) acetonitrile–water mobile phase at 1 mL·min^−1^. The injection volume was 50 µL. Calibration curves were established over 0.5–25.1 mg·L^−1^, showing excellent linearity (R^2^ > 0.999).

### 2.5. Adsorption Experiments

#### 2.5.1. Kinetic Studies

Batch kinetic experiments were conducted by contacting 0.1 g of each biochar (0.01 g for LB_450_–H_3_PO_4_) with 50 mL of BPA solution (*C_0_* = 50 mg·L^−1^) in 100 mL screw-cap Erlenmeyer flasks. The flasks were agitated at 200 rpm and 25 °C in a thermostatic incubator shaker manufactured by New Brunswick Scientific Co., Inc. (Edison, NJ, USA) model G-25. Samples were withdrawn at selected intervals (0.5–92 h), and BPA concentrations were determined by HPLC until equilibrium was reached.

#### 2.5.2. Adsorption Isotherms

To determine the adsorption capacity, 0.1 g of biochar (0.01 g for LB_450_–H_3_PO_4_) was mixed with 50 mL BPA solutions of different initial concentrations (10–100 mg·L^−1^). The mixtures were shaken at 25 °C and 200 rpm until equilibrium. The equilibrium concentration (*C_e_*) and adsorbed amount (*q_e_*, mg·g^−1^) were determined, and adsorption isotherms were constructed.

### 2.6. Effect of Operating Parameters on BPA Adsorption onto LB_450_-H_3_PO_4_

#### 2.6.1. Effect of the Adsorbent Dosage

The effect of the mass-to-volume ratio (*m*/*V*) was investigated by varying the amount of LB_450_–H_3_PO_4_ (0.1–2 g·L^−1^) while keeping the initial BPA concentration (50 mg·L^−1^) constant. The experiments were conducted at 25 °C and 200 rpm for 24 h.

#### 2.6.2. Effect of the Initial BPA Concentration

Adsorption experiments were performed at 25 °C with 0.01 g of LB_450_–H_3_PO_4_ in 50 mL BPA solutions of varying initial concentrations (10–100 mg·L^−1^). After 24 h, the equilibrium concentrations were measured to assess the effect of *C*_0_ on *q_e_*.

#### 2.6.3. Effect of pH

The influence of pH on BPA adsorption was evaluated by adjusting the solution pH from 2 to 12 with 0.1 M HCl or NaOH. Each flask contained 0.01 g of LB_450_–H_3_PO_4_ and 50 mL of 50 mg·L^−1^ BPA solution. The samples were shaken for 24 h at 25 °C and 200 rpm, and the residual BPA concentrations were determined.

#### 2.6.4. Effect of the Temperature

Temperature-dependent studies were carried out at 15, 25, 35, and 45 °C using 0.01 g of LB_450_–H_3_PO_4_ and 50 mL of BPA solution (50 mg·L^−1^). The thermodynamic parameters, Gibbs free energy (ΔG°), enthalpy (ΔH°), and entropy (ΔS°), were determined from the thermodynamic relationships ΔG° = −RT·lnK_c_ and ΔG°= ΔH° − T ΔS°, as well as from the linearized van’t Hoff equation (ln K_c_ versus 1/T).

## 3. Results and Discussion

### 3.1. Characterization of Lignin-Derived Biochars

#### 3.1.1. Elemental Composition and Yield

Table 1 summarizes the elemental composition of the biochars. Increasing the pyrolysis temperature from 110 °C to 450 °C resulted in a gradual increase in the carbon content and a concomitant decrease in the hydrogen and oxygen contents, leading to reduced H/C and O/C ratios. A lower H/C ratio indicates increased aromaticity and carbonization, implying a more condensed aromatic structure and enhanced thermal stability that favor π–π interactions with aromatic molecules such as BPA [4,7]. The decline in O/C ratio reflects the elimination of hydroxyl and carboxyl groups due to the dehydration and decarboxylation reactions typical of lignocellulosic pyrolysis [5].

The chemically activated biochar (LB_450_–H_3_PO_4_) exhibited a lower carbon but higher oxygen content than LB_450_, indicating the successful incorporation of oxygenated functionalities through acid activation. Phosphoric acid acts as a dehydrating and crosslinking agent, promoting phosphate linkage formation that enhances the surface polarity and reactivity [7]. The biochar yield decreased from 96.2% (LB_110_) to 45.9% (LB_450_) with temperature, while chemical activation improved the yield to 77.2%, consistent with the stabilizing effect of H_3_PO_4_ on the carbon framework [14].

#### 3.1.2. FTIR

**Figure 1:**
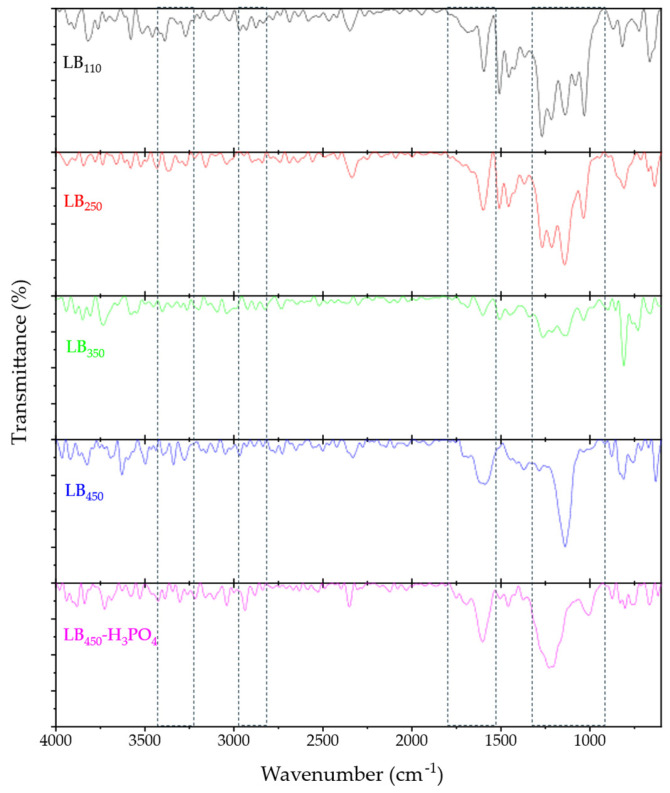
FTIR spectra for the thermally activated (LB_110_, LB_250_, LB_350_, LB_450_ and LB_450_-H_3_PO_4_). The areas marked with dashed lines correspond to the spectral regions showing the most pronounced changes between the different biochars.

The FT-IR spectra of lignin and its derived biochars (Figure 1) clearly illustrate the progressive structural evolution occurring during carbonization and phosphoric acid activation. In the untreated lignin (LB_110_), the broad band at ~3400–3200 cm^−1^ corresponds to O-H stretching of phenolic, aliphatic, and adsorbed-water hydroxyl groups, while the 2950–2800 cm^−1^ region, the weak band observed is attributed to the asymmetric and symmetric C-H stretching of -CH_2_ and –CH_3_ groups, confirming the presence of aliphatic side chains [15,16]. The 1780–1650 cm^−1^ range represents the C=O stretching of carbonyl and carboxylic groups, and the 1600–1500 cm^−1^ region corresponds to aromatic C=C vibrations associated with guaiacyl and syringyl units. The bands at 1470–1410 cm^−1^ arise from -CH_2_/-CH_3_ deformation and in-plane C-H bending of the aromatic ring. The 1280–1200 cm^−1^ range combines C-O, C-C, and aryl-O stretching modes, including overlapping C–C/C–O contributions typical of partially aromatized lignin structures [17]. In the 1150–1000 cm^−1^ region, C-O stretching vibrations of alcohols and ethers predominate, while the 900–800 cm^−1^ range is associated with out-of-plane C-H deformation of substituted aromatic rings.

With increasing temperature (LB_250_-LB_350_), the O-H and C=O bands blue-shift and decrease in intensity, indicating dehydration and the cleavage of conjugated carboxylic groups. The aliphatic C-H stretching bands (2950–2800 cm^−1^) are almost completely lost in these biochars, while the aromatic C=C bands (1600–1500 cm^−1^) become relatively more pronounced, consistent with the progressive aromatization of the material. The appearance of new features in the 900–800 cm^−1^ region indicates the development of more condensed aromatic ring structures [18].

At 450 °C (LB_450_), the spectrum is dominated by aromatic features. The O–H stretching band shifts to ~3300 cm^−1^, reflecting extensive dehydration, while the aliphatic C-H stretching near 2900 cm^−1^ becomes barely detectable, as expected for highly carbonized materials [19]. The band near 1450 cm^−1^ corresponds mainly to aromatic C=C/C-C skeletal deformation, and the broad absorption centered around ~1140 cm^−1^ reflects the cumulative contribution of C-C and C-O stretching within the carbon matrix.

After H_3_PO_4_ activation (LB_450_–H_3_PO_4_), new absorption bands confirm phosphorus incorporation. The most pronounced changes are observed in the bands at ~1200 cm^−1^ and ~1700 cm^−1^, which can be associated with C–O, C=O and P=O vibrations of oxygen-containing and phosphate surface groups. No well-resolved O–H stretching bands are observed in the 3000–3500 cm^−1^ region, which is consistent with the high degree of condensation and the relatively low hydrogen content of the activated biochar. Additional signals in the 870–650 cm^−1^ range (P–O–P and P–O vibrations) confirm the presence of polyphosphate structures [20].

Overall, the blue-shift in the C=O bands, the disappearance of aliphatic C-H stretching, and the broadening of the C-C/C-O region collectively demonstrate progressive thermal deoxygenation, aromatic condensation, and cross-linking during heat treatment [18,19]. Phosphoric acid activation introduces P=O and P–O–C functional groups, enhancing surface acidity and polarity, and providing active sites for BPA adsorption through hydrogen bonding and π–π interactions [5,14,21].

#### 3.1.3. Morphological Analysis

**Figure 2:**
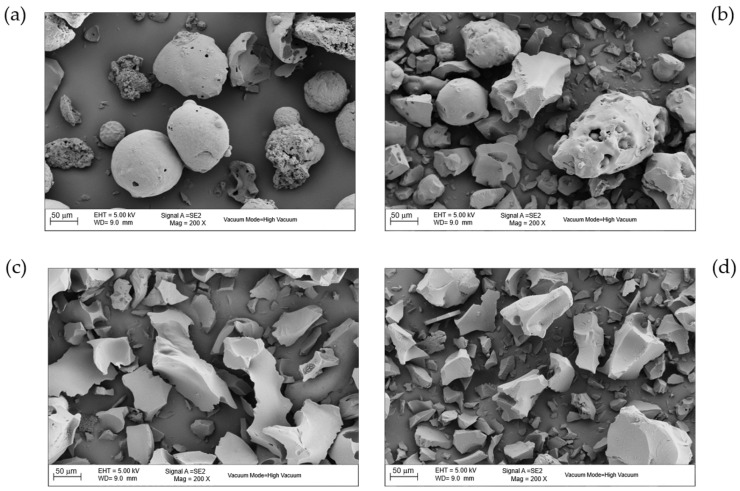
FESEM micrographs of thermally activated biochars at 200× magnification, (**a**) LB_110_, (**b**) LB_205_, (**c**) LB_350_ and (**d**) LB_450_, illustrating the morphological differences induced by the activation temperature.

**Figure 3:**
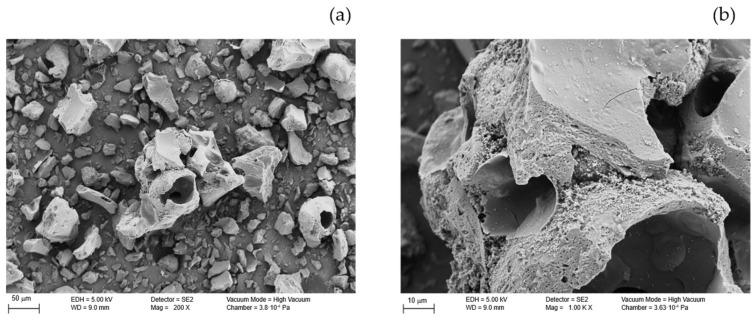
FESEM micrographs of phosphoric acid-activated biochar at 200× (**a**) and 1000× (**b**) magnifications, showing the developed porous surface and morphological changes induced by chemical activation.

The FESEM micrographs (Figure 2) revealed significant morphological changes with temperature. LB_110_ exhibited smooth, spheroidal particles with minimal porosity. Increasing the temperature to 350–450 °C led to the collapse of the initial spheroidal morphology and to a more compact and irregular surface, as evidenced by the loss of spherical particles and the appearance of fractured domains. The chemically activated sample LB_450_–H_3_PO_4_ (Figure 3) displayed a rough, porous surface with well-developed cavities and micropores formed through acid–lignin reactions that release volatile gases such as CO_2_ and H_2_O during carbonization. This morphological transformation aligns with acid-catalyzed crosslinking and dehydration reactions that lead to the formation of micro- and mesoporous structures [13,22].

#### 3.1.4. BET Surface Area and Pore Structure

The BET analysis confirmed that phosphoric acid activation drastically enhanced the surface area and porosity (Table 2). The LB_450_-H_3_PO_4_ sample exhibited a specific surface area of ~522.17 m^2^·g^−1^, representing an almost fortyfold increase compared to the thermally activated LB_450_ (13.03 m^2^·g^−1^). The micropore surface area reached ~349.93 m^2^·g^−1^, while the micropore volume was determined to be 0.18 cm^3^·g^−1^. These results indicate that the H_3_PO_4_ treatment effectively promoted the development of the pore structure in the modified biochar.

This significant increase in porosity can be related to the chemical reactions between H_3_PO_4_ and precursors. The introduction of H_3_PO_4_ may have promoted the cleavage of chemical bonds and formation of crosslinks caused by dehydration, cyclization, and condensation during the pyrolysis process [5,7,13]. Such structural characteristics are essential for maximizing adsorption performance toward aromatic pollutants like BPA.

#### 3.1.5. Point of Zero Charge (PZC)

**Figure 4:**
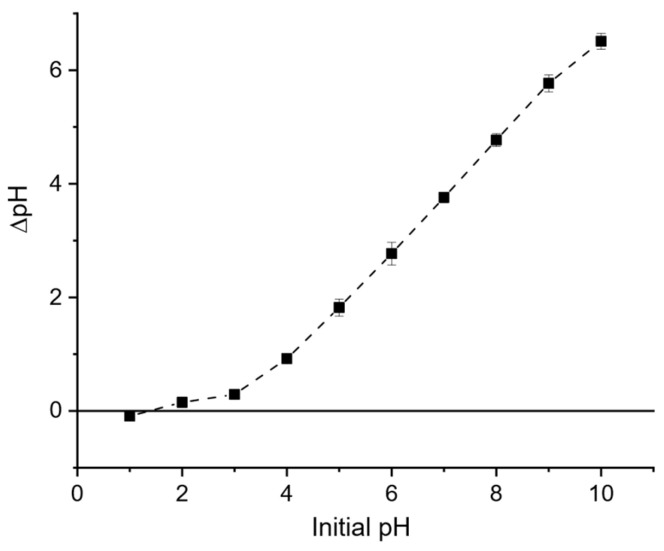
PZC determination by the pH drift method. The point where ΔpH equals zero indicates the point of zero charge (PZC) of the material.

The point of zero charge (PZC) of the acid-treated biochar was determined using the pH drift method. Figure 4 shows the variation in ΔpH as a function of the initial pH. The PZC corresponds to the point at which ΔpH = 0, indicating that the surface of the biochar has no net charge at this pH. As observed in Figure 3, the intersection occurs at approximately pH 1.3, which represents the experimental PZC of the sample LB_450_-H_3_PO_4_.

This remarkably low PZC value reveals that the biochar surface becomes positively charged only under extremely acidic conditions (pH < 1.3), whereas it remains negatively charged in most environmentally relevant pH ranges (pH > 1.3). Such behavior reflects the presence of abundant acidic surface groups (–COOH, –OH) introduced during the acid modification process, which enhance the surface acidity and deprotonation tendency. Similar low PZC values have been reported for acid-treated biochars, where the surface oxidation and removal of basic minerals shift the PZC toward lower pH values [23].

The adsorption of bisphenol A onto this biochar is strongly dependent on the solution pH relative to the PZC. BPA remains mostly in its neutral molecular form below pH 9, favoring π–π interactions, hydrogen bonding, and hydrophobic interactions with the carbonaceous surface. Consequently, the highest adsorption capacity is expected in the acidic to neutral range (pH 4–8), where electrostatic repulsion is minimal and non-electrostatic interactions dominate. At alkaline pH (>9), however, BPA becomes deprotonated (phenolate form) and both the BPA and biochar surface carry negative charges, leading to electrostatic repulsion and reduced adsorption efficiency [14].

### 3.2. Adsorption Behavior of BPA

#### 3.2.1. Adsorption Kinetics

**Figure 5:**
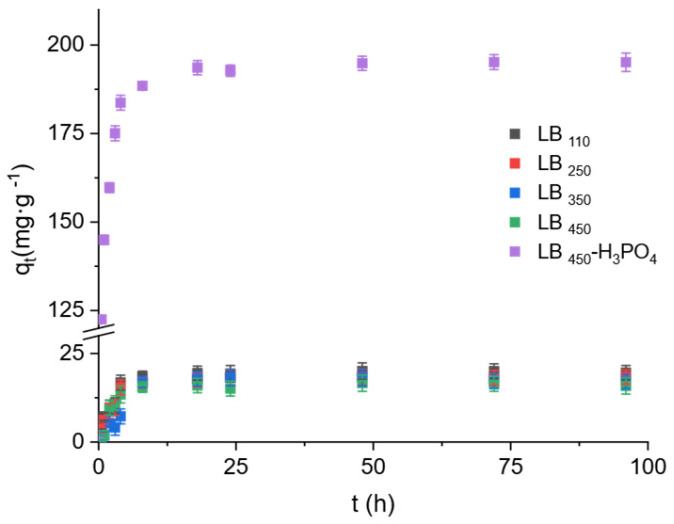
Adsorption kinetics of bisphenol A (BPA) onto thermally activated and acid-modified biochars at 25 °C and an initial concentration of 50 mg·L^−1^. Error bars represent standard deviations of triplicate measurements.

To elucidate the adsorption behavior of BPA on the different biochars, kinetic experiments were performed using thermally activated samples (at 110, 250, 350 and 450 °C) and the acid-modified biochar (LB_450_-H_3_PO_4_). Figure 5 displays the adsorption capacity (*q*ₜ) as a function of contact time for each material. A rapid increase in BPA uptake was observed during the initial stage, achieving 90% of total adsorption within the first 10 h and equilibrium after approximately 24 h. This indicates that many surface sites are readily available at the beginning of the process and become progressively occupied as time advances.

**Figure 6:**
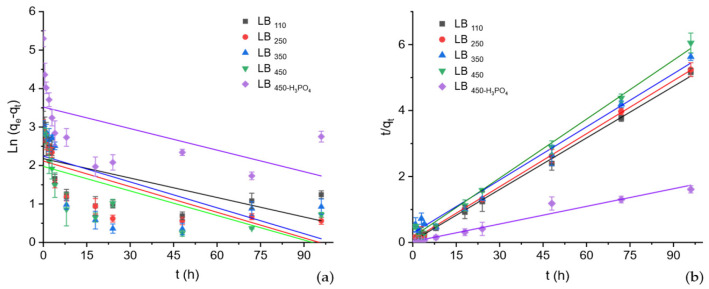
Linearized plots of the adsorption kinetics based on the PFO [log(qₑ − qₜ) vs. t] (**a**) and PSO [t/qₜ vs. t] (**b**) models. The slope and intercept of each linear fit were used to calculate the kinetic constants and equilibrium adsorption capacities (q_e,cal_), and the correlation coefficients (R^2^) were used to assess the fitting quality. Error bars represent standard deviations of triplicate measurements.

Figure 6 presents the linear plots of the pseudo-first order (PFO: $\ln\left(q_{e}-q_{t}\right)=\ln q_{e}-k_{1}t$) and pseudo-second order (PSO: $\frac{t}{q_{t}}=\frac{1}{k_{2}q_{e}^{2}}+\frac{t}{q_{e}}$) kinetic models for BPA adsorption into thermally and acid-activated biochars and the kinetic parameters are summarized in Table 3. The experimental data for all biochars fit the PSO model much better than the PFO model, as evidenced by the higher correlation coefficients (*R^2^* ≥ 0.985) and the close agreement between the experimental (*q_e,exp_*) and calculated (*q_e,cal_*) adsorption capacities. In contrast, the PFO model showed poor correlation (*R^2^* < 0.55) and significant deviations in the calculated *qₑ* values, indicating that this model does not adequately describe the adsorption process.

The PSO rate constant (k_2_) and equilibrium adsorption capacity (*q_e,cal_*) followed the order LB_450_-H_3_PO_4_ ≫ LB_110_ > LB_250_ > LB_350_ > LB_450_, confirming the remarkable enhancement in adsorption performance after acid activation. The acid-treated biochar (LB_450_-H_3_PO_4_) exhibited an exceptionally high adsorption capacity (*q_e,exp_* = 193.48 ± 6.50 mg·g^−1^), nearly ten times greater than that of the thermally activated samples (16–20 mg·g^−1^). This result reflects the profound influence of acid modification on the surface chemistry, leading to the introduction of oxygenated functional groups and improved pore accessibility for BPA molecules [13].

The excellent agreement of the experimental data with the PSO model suggests that the adsorption of BPA on both thermally and acid-activated biochars is mainly governed by chemisorption mechanisms, involving π–π electron donor–acceptor interactions and hydrogen bonding between BPA and surface oxygen-containing groups. These findings are consistent with previous reports indicating that acid activation enhances the chemical affinity and electron density on biochar surfaces, thereby facilitating stronger adsorbate–adsorbent interactions [1,14].

#### 3.2.2. Adsorption Isotherms

To further elucidate the adsorption mechanism and surface characteristics of the biochars, equilibrium adsorption experiments were conducted at different initial BPA concentrations. The resulting adsorption isotherms describe the relationship between the equilibrium concentration of BPA in the solution (*C_e_*) and the amount adsorbed on the biochar surface (*q_e_*). These data provide key insights into the affinity of the adsorbent for BPA molecules and the interaction between them.

**Figure 7:**
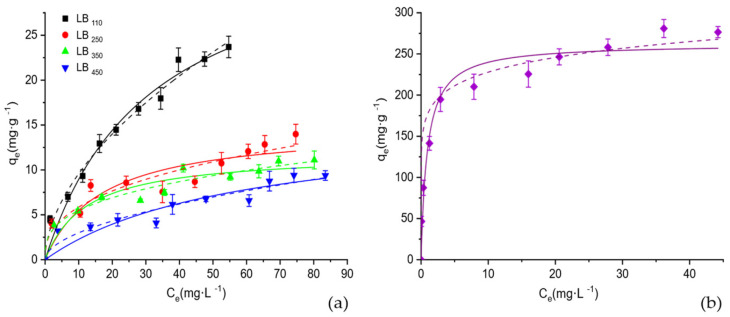
Adsorption isotherms of bisphenol A (BPA) on thermally activated (**a**) and acid-modified (**b**) biochars at 25°C. Experimental data (symbols) and model fittings according to the Langmuir (solid line) and Freundlich (dash line) isotherm models are shown. Error bars represent standard deviations of triplicate measurements.

Figure 7 illustrates the adsorption isotherms obtained for both the thermally activated and acid-modified biochars. A clear difference can be observed between the materials: the acid-activated biochar (LB_450_-H_3_PO_4_) exhibits a much steeper initial slope and higher plateau value, indicating a substantially greater adsorption capacity compared with the thermally activated samples. This behavior reflects the enhanced surface functionality and porosity induced by the acid treatment.

To quantitatively interpret the equilibrium data, the experimental results were fitted using the non-linear forms of the Langmuir and Freundlich isotherm models. The Langmuir model assumes monolayer adsorption on a homogeneous surface with identical binding sites and is expressed as $q_{e}=\frac{q_{\max}K_{L}C_{e}}{1+K_{L}C_{e}}$, where $q_{m}$(mg·g^−1^) represents the maximum adsorption capacity and $K_{L}$(L·mg^−1^) is the Langmuir constant related to the affinity between the adsorbate and the adsorbent. The dimensionless separation factor, $R_{L}=\frac{1}{1+K_{L}C_{0}}$, where $C_{0}$ is the initial solute concentration, was also calculated to evaluate the favorability of the adsorption process ($0<R_{L}<1$ indicates favorable adsorption). In contrast, the Freundlich model describes adsorption on a heterogeneous surface with sites of different energies and is given by $q_{e}=K_{F}C_{e}^{1/n}$, where $K_{F}({\mathrm{mg}\cdot g}^{-1})({L\cdot \mathrm{mg}}^{-1}{)}^{1/n}$ and 1/n are the Freundlich constants related to adsorption capacity and surface heterogeneity, respectively. The fitting parameters obtained from both models are summarized in Table 4.

Both models adequately described the experimental data, as indicated by the high correlation coefficients (*R^2^* > 0.85). However, for most samples, the Langmuir model provided a slightly better fit, suggesting that BPA adsorption mainly occurs through monolayer coverage on relatively homogeneous active sites.

Among all samples, the acid-activated biochar (LB_450_-H_3_PO_4_) exhibited an exceptionally high maximum adsorption capacity (*q_m_* = 262.28 ± 14.3 mg·g^−1^), significantly exceeding those reported for other lignin-derived or agricultural biochars (<100 mg·g^−1^) [24,25], and which is nearly an order of magnitude higher than those of the thermally activated biochars (*q_m_* ≈ 12–37 mg·g^−1^). The very low separation factor (*R_L_* ≈ 0.01) further indicates a highly favorable adsorption process. This remarkable enhancement can be attributed to the acid treatment, which introduces oxygenated functional groups and increases the surface polarity and porosity, thereby improving the affinity toward BPA molecules. These results clearly indicate that the outstanding adsorption enhancement of the acid-activated biochar is primarily controlled by surface chemistry, namely, the presence of oxygenated and phosphate functional groups and the associated surface charge, rather than by surface area alone.

In contrast, the thermally activated biochars (LB_110_–LB_450_) showed much lower adsorption capacities and smaller K_L_ values, implying weaker interactions between BPA and the less functionalized carbon surfaces. The Freundlich constants also support these observations: the acid-treated biochar exhibits the highest *K_F_* (128.43 ± 2.61 L·mg^−1^) and the lowest *n* (0.21 ± 0.05), consistent with the strong adsorption affinity on a heterogeneous surface containing sites of varying energy.

Overall, these results demonstrate that acid activation dramatically enhances both the adsorption capacity and affinity of biochar toward BPA, confirming that surface chemistry, rather than only surface area, plays a decisive role in determining adsorption performance.

#### 3.2.3. Proposed Adsorption Mechanism of BPA on the Biochars

Based on the characterization results and the kinetic and isotherm analyses, the adsorption of BPA onto the studied biochars involves a combination of physical and chemical interactions that are strongly influenced by the surface chemistry and activation method. The kinetic data showed that all samples followed the pseudo-second-order model, suggesting that chemisorption predominates through valence forces involving electron sharing or exchange between the adsorbate and the adsorbent surface.

The very low point of zero charge (PZC = 1.3) of the acid-treated biochar indicates that its surface is negatively charged under most environmental pH conditions (pH > 1.3). Since BPA remains in a neutral molecular form at pH below 9, electrostatic repulsion is minimal, allowing other mechanisms, particularly π–π electron donor–acceptor (EDA) interactions, hydrogen bonding, and hydrophobic partitioning, to dominate the adsorption process.

The enhanced adsorption capacity of the acid-activated biochar (LB_450_-H_3_PO_4_), as revealed by the Langmuir model (*q_m_* = 262.28 ± 14.3 mg·g^−1^), can be attributed to the combined effect of increased surface functionalization and porosity induced by phosphoric acid treatment. The introduction of oxygen-containing groups (–COOH, –OH, –C=O) promotes hydrogen bonding and π–π stacking between the aromatic rings of BPA and the conjugated carbon structures on the biochar surface [26,27]. In addition, the removal of inorganic minerals during acid activation exposes more accessible adsorption sites, facilitating the diffusion of BPA molecules into the internal pores.

**Figure 8:**
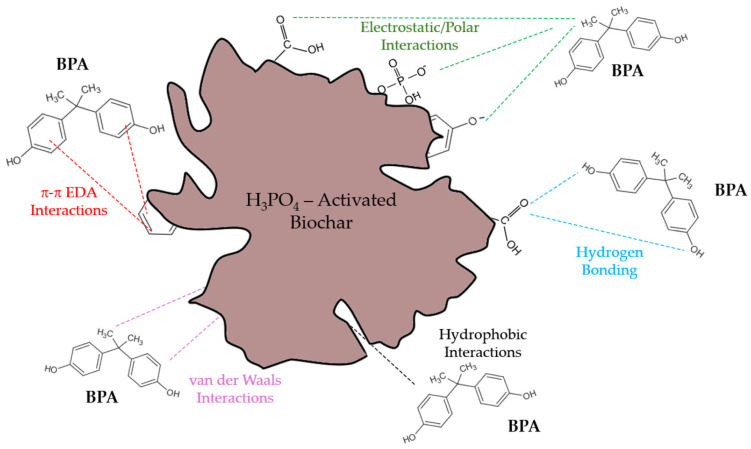
Schematic representation of the main interactions for BPA adsorption onto acid-activated biochar (LB_450_–H_3_PO_4_).

For the thermally activated biochars, the lower adsorption capacities (*q_m_* = 12–37 mg·g^−1^) reflect their more hydrophobic and less functionalized nature, where adsorption is mainly governed by weaker van der Waals forces and limited π–π interactions (Figure 8).

Therefore, the overall adsorption mechanism of BPA onto biochar can be described as a multistep process involving (i) rapid surface adsorption driven by π–π and hydrogen-bond interactions, (ii) gradual pore diffusion, and (iii) equilibrium governed by chemisorption on oxygenated functional groups. Acid activation clearly enhances each of these stages by increasing both the density and reactivity of the surface sites, leading to superior adsorption performance compared with thermally activated biochars.

It is also worth noting that BPA adsorption proceeds through non-covalent interactions and therefore does not induce chemical or structural modification of the biochar. For this reason, the mechanistic interpretation relies on the pre-adsorption characterization together with the kinetic, isotherm, and thermodynamic analyses, which consistently support the proposed multistep adsorption pathway.

The adsorption behavior of BPA on the acid-activated biochar is mainly governed by non-covalent interactions acting synergistically (Figure 8). Among these, hydrophobic effects and π–π electron donor–acceptor interactions (π-π EDA) between the aromatic rings of BPA and the conjugated domains of the biochar are expected to play a dominant role, as they typically promote the exclusion of structured water and favor adsorption through entropy gain. Hydrogen bonding between the hydroxyl groups of BPA and the oxygenated or phosphate surface groups provides additional stabilization of the adsorbed molecules, while electrostatic interactions modulate the overall process depending on solution pH and the low point of zero charge (PZC = 1.3). Under acidic to neutral conditions, electrostatic repulsion is negligible, whereas under alkaline conditions, deprotonation of BPA and the negatively charged surface lead to repulsive forces that reduce adsorption efficiency. These combined interactions explain the high affinity of the acid-activated biochar toward BPA.

### 3.3. Effect of Operational Parameters on BPA Adsorption Using Acid-Activated Biochar

After identifying the acid-activated biochar (LB_450_-H_3_PO_4_) as the most efficient adsorbent for BPA, additional experiments were performed to evaluate the influence of key operational parameters on the adsorption process. This analysis aimed to optimize the conditions that maximize BPA removal and to better understand the mechanisms governing adsorption under different environmental scenarios.

**Figure 9:**
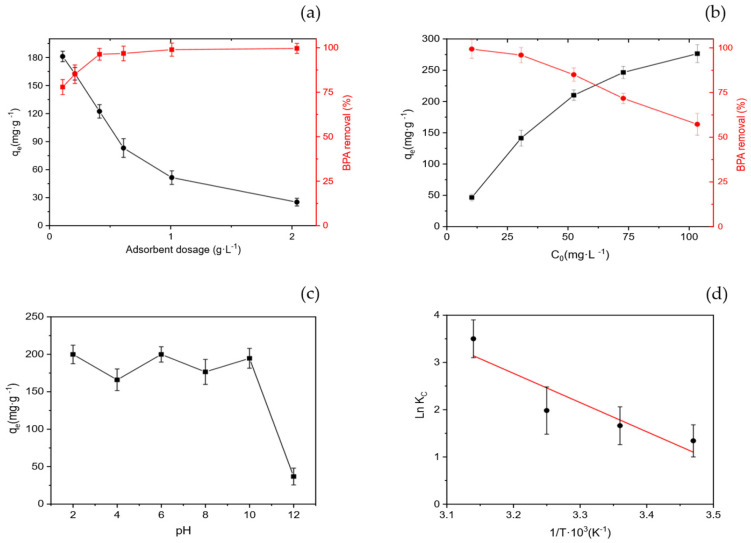
Effect of operational parameters on BPA adsorption by acid-activated biochar (LB_450_–H_3_PO_4_): (**a**) adsorbent dosage, (**b**) initial BPA concentration, (**c**) pH, and (**d**) temperature. Investigating these factors exclusively for the acid-activated biochar is justified by its outstanding adsorption performance (*q_m_* = 262.28 ± 14.3 mg·g^−1^) compared with the thermally activated samples. Moreover, the presence of abundant oxygen-containing functional groups and a high surface area make this material particularly sensitive to changes in solution chemistry, allowing a more accurate evaluation of the adsorption behavior under realistic operating conditions. Error bars represent standard deviations of triplicate measurements.

The studied parameters included solution pH, initial BPA concentration, adsorbent dosage, and temperature, all of which significantly affect adsorption efficiency by altering both the surface charge of the adsorbent and the chemical speciation of BPA. Figure 9 presents the variation in adsorption capacity and removal percentage as a function of these parameters

#### 3.3.1. Effect of Adsorbent Dose

As shown in Figure 9a, the removal efficiency of BPA increased markedly from 77.9% to 99.6% as the adsorbent dosage rose from 0.1 to 2.0 g·L^−1^. This improvement is attributed to the greater availability of active sites and surface area for adsorption at higher biochar dosages. However, the equilibrium adsorption capacity (*q_e_*) decreased from approximately 181 to 25 mg·g^−1^, which can be explained by the overlapping of adsorption sites and particle aggregation at higher concentrations, reducing the effective surface area per unit mass. This trend has been widely reported for phosphoric-acid-activated biochars, where excessive dosage leads to site interference and reduced mass-normalized uptake [5,13]. Based on these results, the optimal m/V ratio for BPA removal was determined to be 0.2 g·L^−1^.

#### 3.3.2. Effect of Initial BPA Concentration

As depicted in Figure 9b, increasing the initial BPA concentration from 10 to 100 mg·L^−1^ resulted in a significant decrease in removal efficiency, from 99.3% to 57.2%, while the adsorption capacity (*q_e_*) increased substantially from approximately 46 to 277 mg·g^−1^. The increase in *q_e_* reflects a stronger driving force for mass transfer at higher solute concentrations, enhancing the probability of BPA molecules reaching and occupying the available active sites on the biochar surface. The subsequent reduction in removal percentage at high concentrations indicates saturation of adsorption sites, consistent with Langmuir-type monolayer adsorption behavior [7]. Similar trends have been observed for the adsorption of phenolic compounds onto acid-activated biochars [21].

#### 3.3.3. Effect of Solution pH

The effect of solution pH on BPA adsorption is shown in Figure 9c. The adsorption capacity remained nearly constant between pH 2 and 10, but a sharp decline occurred at pH > 10. Considering the point of zero charge (PZC = 1.3) of the biochar and the pK_a_ values of BPA (9.6 and 10.2), both the adsorbent and BPA become negatively charged in highly alkaline media, resulting in electrostatic repulsion that reduces adsorption. Below this pH range, BPA is predominantly neutral, and adsorption is driven by π–π electron donor–acceptor interactions and hydrogen bonding between BPA and oxygenated surface groups on the biochar (Figure 8). These interactions explain the strong affinity observed under acidic to neutral conditions, consistent with previous studies on phosphoric-acid-activated biochars [3,13].

#### 3.3.4. Effect of Temperature and Thermodynamics

Thermodynamic parameters (Table 5) confirm that the adsorption of BPA onto the acid-activated biochar is spontaneous throughout the entire temperature range studied, as evidenced by the negative Gibbs free energy values (ΔG° = −3.21 to −9.25 kJ·mol^−1^). The process is endothermic (ΔH° = 51.14 kJ·mol^−1^; negative slope in Figure 9d) and accompanied by a large positive entropy change (ΔS° = 187.48 J·mol^−1^·K^−1^), indicating that spontaneity is entropy-driven rather than enthalpy-driven. This high ΔS° reflects a significant increase in disorder at the solid–liquid interface, primarily associated with desolvation of BPA molecules and the release of structured water from the biochar surface. Such an entropy gain is characteristic of adsorption dominated by hydrophobic interactions and π-π stacking, where exclusion of water from aromatic domains increases system randomness. In contrast, enthalpy-driven adsorption (ΔH° < 0) typically corresponds to hydrogen bonding or electrostatic interactions, which are exothermic but lead to interfacial ordering. Therefore, in this system, the positive ΔH° and high ΔS° values indicate that hydrophobic and π-π interactions are the principal driving forces, while hydrogen bonding plays a secondary role in stabilizing the adsorbed BPA species. These thermodynamic findings are consistent with the interaction mechanisms previously discussed, confirming that adsorption is mainly governed by hydrophobic and π-π interactions, complemented by hydrogen bonding and pH-dependent electrostatic effects.

## 4. Conclusions

The comprehensive evaluation of thermally and acid-activated biochars demonstrated that surface chemistry, charge properties, and pore structure are the main factors governing the adsorption of bisphenol A (BPA). Characterization analyses confirmed that phosphoric acid activation significantly enhanced the surface area, pore development, and oxygen/phosphate functionalization of the biochar, resulting in a markedly lower point of zero charge (PZC = 1.3) and improved surface acidity. These modifications provided a larger number of reactive sites for BPA interaction through π–π stacking, hydrogen bonding, and chemisorption on oxygenated groups.

Kinetic studies revealed that BPA adsorption followed the pseudo-second-order model, indicating a chemisorption-controlled process. Equilibrium data were best fitted by the Langmuir isotherm, confirming monolayer adsorption on homogeneous active sites with a maximum capacity of 262.28 ± 14.3 mg·g^−1^ for the acid-activated biochar (LB_450_-H_3_PO_4_). Thermodynamic parameters (ΔG° < 0, ΔH° = 51.14 ± 15.7 kJ mol^−1^, ΔS° > 0) showed that adsorption is spontaneous, endothermic, and favored at higher temperatures.

The effect of operational parameters demonstrated that optimal removal of BPA occurs under acidic to neutral pH, at a moderate adsorbent dosage (0.2 g·L^−1^) and elevated temperature, which enhances the diffusion and interaction of BPA molecules with active surface sites. In contrast, thermally activated biochars exhibited lower adsorption capacities due to their limited surface functionality and higher hydrophobicity.

Overall, phosphoric acid activation effectively transforms biochar into a highly efficient, stable, and environmentally sustainable adsorbent for the removal of BPA and related phenolic contaminants from aqueous media. The results confirm that the remarkable improvement in adsorption capacity arises mainly from modifications in surface chemistry, particularly the introduction of oxygenated and phosphate groups and the resulting surface charge, rather than from surface area development alone. These insights provide a rational basis for designing next-generation, lignin-derived biochars optimized for pollutant adsorption.

## Figures and Tables

**Table 1:** Elemental analysis of the on thermally and acid-activated biochars.

Biochar	N [%]	C [%]	H [%]	S [%]	O [%] *	H/C	O/C	Yield (%)
LB_110_	0.46	61.18	5.33	1.70	31.33	0.08	0.51	96.18
LB_250_	0.37	66.84	4.12	1.43	27.24	0.06	0.40	66.26
LB_350_	0.49	67.87	4.75	1.47	25.42	0.06	0.37	48.33
LB_450_	0.65	76.66	3.18	1.45	18.05	0.04	0.23	45.87
LB_450_-H_3_PO_4_	0.37	65.31	3.61	0.38	30.33	0.05	0.46	77.23

* obtained by difference.

**Table 2:** BET surface area for the thermally and acid-activated biochars.

Biochar	Surface Area BET (m^2^·g^−1^)
LB_110_	1.46 ± 0.11
LB_250_	0.31 ± 0.06
LB_350_	0.44 ± 0.07
LB_450_	13.03 ± 1.35
LB_450_-H_3_PO_4_	522.17 ± 2.27

**Table 3:** Kinetic parameters for BPA adsorption on thermally and acid-activated biochars.

Biochar	*q_e,exp_* (mg∙g^−1^)	Pseudo-First Order (PFO) *			Pseudo-Second Order (PSO) *		
		*q_e,cal_* (mg∙g^−1^)	*k_1_*·10^2^ (h^−1^)	*R^2^*	*q_e,cal_* (mg∙g^−1^)	*k_2_*·10^2^ (g∙mg^−1^∙h^−1^)	*R^2^*
LB_110_	19.74 ± 0.35	8.87 ± 2.27	1.6 ± 0.67	0.389	19.15 ± 0.29	6.6 ± 1.8	0.999
LB_250_	18.24 ± 0.09	8.33 ± 2.05	2.2 ± 0.64	0.542	18.60 ± 0.14	3.7 ± 0.7	0.999
LB_350_	17.63 ± 0.61	9.47 ± 3.03	2.2 ± 0.84	0.411	18.41 ± 0.71	1.1 ± 0.3	0.985
LB_450_	16.01 ± 0.68	7.24 ± 1.93	2.1 ± 0.67	0.482	16.68 ± 0.47	2.4 ± 0.9	0.992
LB_450_-H_3_PO_4_	193.48 ± 6.50	34.26 ± 11.60	1.7 ± 0.88	0.282	189.02 ± 38.22	47.8 ± 8.8	0.999

* Errors correspond to the uncertainties obtained from the fitting of triplicate experimental data to PFO and PSO kinetic models. *q_e,exp_* (expressed as mean value ± SD) and *q_e,cal_* are the experimental and calculated equilibrium adsorption capacities, and k_1_ and k_2_ denote the PFO and PSO rate constants, respectively.

**Table 4:** Parameters of the Langmuir and Freundlich isotherms for the thermally and acid-activated biochars *.

Biochar	Langmuir				Freundlich		
	*K_L_*·10^2^ (L∙mg^−1^)	*q_m_* (mg∙g^−1^)	*R_L_*	*R^2^*	*n*	*K_F_* (L∙mg^−1^)	*R^2^*
LB_110_	3.2 ± 0.2	37.14 ± 0.7	0.24 ± 0.06	0.977	0.54 ± 0.20	2.70 ± 0.30	0.981
LB_250_	5.8 ± 0.6	14.84 ± 1.3	0.14 ± 0.07	0.855	0.38 ± 0.11	2.40 ± 0.21	0.855
LB_350_	8.2 ± 0.7	11.87 ± 0.8	0.11 ± 0.05	0.909	0.33 ± 0.07	2.53 ± 0.21	0.908
LB_450_	1.7 ± 0.2	15.29 ± 1.2	0.37 ± 0.10	0.876	0.52 ± 0.13	0.89 ± 0.10	0.865
LB_450_-H_3_PO_4_	107 ± 12	262.28 ± 14.3	0.01 ± 0.01	0.964	0.21 ± 0.05	128.43 ± 2.61	0.974

* Errors correspond to the uncertainties obtained from the fitting of triplicate experimental data to the Langmuir and Freundlich models. *R_L_* is the separation factor.

**Table 5:** Thermodynamic parameters for BPA adsorption onto acid-activated biochar (LB_450_–H_3_PO_4_).

Temperature (K)	ΔGº (kJ·mol^−1^)	ΔHº (kJ·mol^−1^)	ΔSº (J·mol^−1^·K^−1^)
288	−3.21 ± 0.81	51.14 ± 15.7	187.48 ± 51.57
298	−4.11 ± 0.99		
308	−5.07 ± 1.12		
318	−9.25 ± 1.06		

## Data Availability

The original contributions presented in this study are included in the article. Further inquiries can be directed to the corresponding author.
